# Annual incidence rates of herpes zoster among an immunocompetent population in the United States

**DOI:** 10.1186/s12879-015-1262-8

**Published:** 2015-11-06

**Authors:** Barbara H. Johnson, Liisa Palmer, Justin Gatwood, Gregory Lenhart, Kosuke Kawai, Camilo J. Acosta

**Affiliations:** Outcomes Research, Truven Health Analytics, Ann Arbor, MI USA; Vaccines, Center for Observational and Real-World Evidence (CORE), Merck & Co., Inc., West Point, PA USA

**Keywords:** Herpes zoster, Incidence rates, Immunocompetent, Vaccine

## Abstract

**Background:**

Herpes zoster (HZ), also known as shingles, is a painful and commonly occurring condition in the United States. In spite of a universally recommended vaccine for use in immunocompetent adults aged 60 years and older, HZ continues to impact the American public, and a better understanding of its current incidence is needed. The objective of the current study is to estimate the overall and age- and gender-specific incidence rates (IRs) of HZ among an immunocompetent US population in 2011 following availability of a vaccine.

**Methods:**

Claims data from the Truven Health MarketScan® Research databases between 01/01/2011 and 12/31/2011 were extracted. Immunocompetent adult patients, enrolled as of January 1, 2011 were analyzed. The denominator was defined as eligible subjects who were immunocompetent, had no evidence of zoster vaccination, and no diagnosis of HZ (International Classification of Diseases, Ninth Revision, Clinical Modification diagnosis code 053.xx) in the 90 days prior to January 1, 2011. Subjects contributed person-days to the denominator until the occurrence of one of the following events: end of continuous enrollment in the database, a claim for zoster vaccination, diagnosis of HZ or end of the observation period (December 31, 2011). The numerator was defined as enrollees within the denominator file exhibiting evidence of HZ. Annual IRs were calculated for the entire population in the database as well as by gender and age group; standardized IRs were also produced using the 2010 US Census data.

**Results:**

The overall annual IR of HZ across all ages was 4.47 per 1000 person-years (95 % confidence interval [CI]: 4.44–4.50) which monotonically increased with age from 0.86 (95 % CI: 0.84–0.88) for those aged ≤19 to 12.78 (95 % CI: 12.49–13.07) for patients ≥80 years. The IR was 8.46 (95 % CI: 8.39–8.52) among adults ≥50 years and 10.46 (95 % CI: 10.35–10.56) among those aged ≥60 years. Women compared to men had higher HZ incidence (5.25, 95 % CI: 5.21–5.29 vs. 3.66, 95 % CI: 3.62–3.69) and this was seen across all age groups. When adjusted for age and gender using 2010 US Census data, the annual IR was 4.63 per 1000 person-years (95 % CI: 4.61–4.66).

**Conclusions:**

Despite the availability of a vaccine, HZ remains common among immunocompetent adults in the US with incidence rates of HZ observed to increase with age and be higher in women than men.

**Electronic supplementary material:**

The online version of this article (doi:10.1186/s12879-015-1262-8) contains supplementary material, which is available to authorized users.

## Background

Herpes zoster (HZ), or shingles, is caused by reactivation of the varicella zoster virus (VZV) and mainly affects the elderly population [1–3]. In the United States, over 99 % of adults have serological evidence of VZV infection and are susceptible to HZ [4], and the individual lifetime risk of HZ is approximately 30 % [5]. It is estimated that approximately 1 million new cases of HZ are diagnosed each year. While susceptibility to HZ increases with advancing age, the rate of incidence significantly increases among patients aged >50 years, with a drastic rise in the rate thereafter [2, 6]. The incidence rate of HZ ranged between three and five per 1,000 person-years from prior studies in the US and other countries, depending on the population studied and immunocompetency of subjects [5, 7–10]. However, estimates are prior to the introduction of a zoster vaccine in the United States.

Zostavax^®^, a live attenuated zoster vaccine approved in the US for the prevention of HZ in immunocompetent adults 50 years of age and older, is recommended by the US Centers for Disease Control and Prevention’s Advisory Committee on Immunization Practices (ACIP) for use among immunocompetent adults aged 60 years and older [11]. In a large randomized clinical trial, zoster vaccine was shown to reduce the incidence of HZ by 51.3 % and the incidence of postherpetic neuralgia (PHN) by 66.5 %, in immunocompetent adults 60 years of age and older [6]. In addition, the Zostavax Efficacy and Safety Trial (ZEST) demonstrated that zoster vaccine reduced the risk of HZ in adults 50 to 59 [12]. The economic value of the zoster vaccine has been reviewed and synthesized elsewhere [13].

In spite of vaccine availability, HZ continues to pose a significant burden in the US. Despite ACIP recommendations, in 2011 only 15.8 % of adults aged ≥60 years reported ever receiving the zoster vaccine [14]. Previous studies found that the incidence rate of HZ has changed over time, and updated data on age-specific and sex-specific incidence rates using a nationwide database is important to understand the population at risk of HZ. The objective of the current study is to estimate the overall and age- and sex-specific incidence rates of HZ among immunocompetent US adults in 2011 following availability of a vaccine.

## Methods

### Data source

This retrospective, observational cohort study was conducted using administrative claims data from two Truven Health MarketScan^®^ Research Databases – the Commercial Claims and Encounters and the Medicare Supplemental and Coordination of Benefits—for the period January 1, 2011 through December 31, 2011. Both databases include complete longitudinal records of inpatient services, outpatient services, long-term care, and prescription drug claims covered under a variety of fee-for-service and managed care health plans, including exclusive provider organizations, preferred provider organizations, point of service plans, indemnity plans, and health maintenance organizations. The MarketScan Research database records are de-identified and compliant with US patient confidentiality requirements, including the Health Insurance Portability and Accountability Act 1996. Permissions were not needed to utilize these data. Since this study used only de-identified patient records and did not involve the collection, use, or transmittal of individually identifiable data, Institutional Review Board approval was not necessary.

### Patient selection

Patients with and without HZ (International Classification of Diseases, Ninth Revision, Clinical Modification [ICD-9-CM] diagnosis code 053.xx), enrolled as of January 1, 2011, having no claims for HZ or evidence of receiving shingles vaccination in the 90 days prior to January 1, 2011, and meeting criteria for immunocompetent status were included. A series of selected ICD-9-CM diagnoses, procedures and treatment criteria, informed by the Centers for Disease Control and Prevention [11] were used to determine the immunocompetent status of each patient. Patients were excluded if they had ICD-9-CM diagnosis or procedure codes for hematologic malignancy, solid tumor malignancy, human immunodeficiency virus, chronic renal failure, nephrotic syndrome, and other select immunocompromising conditions. Patients were also excluded if they had evidence of organ transplantation, procedures indicating injection or infusion of cancer chemotherapeutic substance, immunotherapy as antineoplastic agent, poisoning by antineoplastic and immunosuppressive drugs, or treatment with chemotherapy, radiation therapy, corticosteroids, tumor necrosis factor inhibitors, protease inhibitors, reverse transcriptase inhibitors, azathioprine, cyclosporine, or tacrolimus.

### Annual incidence rate

The annual incidence rates of HZ were calculated for the entire population observed in the database as well as by gender and age group (<19, 20–29, 30–39, 40–49, 50–59, 60–69, 70–79, 80+). Standardized incidence rates were also produced using the 2010 US Census data to produce overall and gender-specific rates by age group [15]. In addition, rates were reported for those aged ≥50 and ≥60 years.

### Statistical analysis

Descriptive analyses were conducted to determine the demographic characteristics of the study cohort. Annual incidence rates of HZ in the immunocompetent were estimated by dividing the total unique number of incident HZ patients observed in the reporting year by the total number of person-years in the reporting year. The calculations for the denominator and numerator were performed as described previously [16]. The denominator was defined as eligible subjects who were immunocompetent, had no evidence of shingles vaccination, and no diagnosis of HZ in the 90 days prior to January 1, 2011. Subjects contributed person-days to the denominator until the occurrence of one of the following events: end of continuous enrollment in the database, a claim for zoster vaccine, diagnosis of HZ, or end of the observation period (December 31, 2011). Rates of HZ were presented per 1,000 person-years, and the direct standardized rate and 95 % confidence intervals were calculated by Chiang’s normal approximation to Poisson rate sums [17]. A *p*-value of <0.05 was considered statistically significant.

## Results

A total of 113,861 incident cases of HZ were observed in the database during the study period. Demographic characteristics of the study cohort are shown in Table 1. The mean population age was 52 (standard deviation: 17.8) years, with the majority aged ≥50 years (60 %), and females comprising 60 % of the study population. Over a third of patients resided in the south region of the United States (36 %) and the majority was covered by commercial insurance (79.1 %) (Table 1).

**Table 1:** Demographic Characteristics of Incident Herpes Zoster Population

	All HZ Patients	Total Denominator
Demographic Characteristics	*N* = 113, 861	*N* = 27,616,373
Mean age, year (SD)	52.0 (17.8)	36.9 (21.0)
Age Group, *n* (%)		
<50	45,588 (40)	18,972,479 (69)
50–59	29,266 (26)	4,620,496 (17)
60–64	14,959 (13)	1,866,686 (7)
65–69	6,636 (6)	674,081 (2)
70–79	10,231 (9)	884,385 (3)
80+	7,181, (6)	598,246 (2)
Gender, *n* (%)		
Male	45,457 (40)	13,496,731 (49)
Female	68,404 (60)	14,119,642 (51)
Health Plan, *n* (%)		
Comprehensive/Indemnity	13,128 (12)	1,366,332 (5)
EPO/PPO	62,100 (55)	15,751,867 (57)
POS/POS w/capitation	7,806 (7)	2,128,553 (8)
HMO	16,430 (14)	4,235,960 (15)
CDHP/HDHP	6,905 (6)	2,109,739 (8)
Missing/Unknown	7,492 (7)	2,023,922 (7)
Primary Insurance, *n* (%)		
Commercial	89,495 (79)	25,416,170 (92)
Medicare	24,366 (21)	2,200,203 (8)
Geographic Region, *n* (%)		
Northeast	20,268 (18)	5,113,594 (19)
North Central	29,490 (26)	6,765,658 (25)
South	40,575 (36)	9,976,789 (36)
West	22,653 (20)	5,471,074 (20)
Unknown Region	875 (1)	289,258 (1)
Employee classification, *n* (%)		
Employee, salaried	11,441 (10)	2,246,437 (8)
Employee, hourly	14,944 (13)	2,524,118 (9)
Employee, unknown	48,419 (43)	9,006,547 (33)
Spouse or dependent	39,057 (34)	13,839,271 (50)
Employee Relationship, *n* (%)		
Employee	74,804 (66)	13,777,102 (50)
Spouse	30,900 (27)	5,415,157 (20)
Child/Other	8,157 (7)	8,424,114 (31)

*HMO* health maintenance organization, *POS* point-of-service, *PPO/EPO* preferred provider organization/exclusive provider organization, *CDHP/HDHP* consumer-driven health plan/high-deductible health plan

The overall annual incidence rate of HZ, across all ages of the study population, was 4.47 per 1,000 person-years (95 % confidence interval [CI]: 4.44–4.50) with higher rates observed among women than men (5.25, 95 % CI: 5.21–5.29 vs. 3.66, 95 % CI: 3.62–3.69), respectively) across all age groups. The rate of HZ increased monotonically with age, ranging from 0.86 (95 % CI: 0.84–0.88) for those aged ≤19 to 12.78 (95 % CI: 12.49–13.07) for immunocompetent HZ patients aged 80 and older (see Additional file 1). When the raw annual incidence rates of HZ across all ages were adjusted for age and sex using 2010 US Census data, the overall rate was 4.63 per 1,000 person-years (95 % CI: 4.61, 4.66), with higher rates observed among women than men (5.52, 95 % CI: 5.45–5.53 vs. 3.66, 95 % CI: 3.62–3.79, respectively).

When incidence rates of HZ were examined for combined cohorts across age groups (i.e. those aged 50 and older and those 60 and older), the incidence rate increased from 8.46 (95 % CI: 8.39–8.52) among adults 50 years and older to 10.46 (95 % CI: 10.35–10.56) among those aged 60 years and older. Similarly, rates for males and females followed a nearly identical trend, increasing from 6.71 (95 % CI: 6.63–6.80) to 8.73 (95 % CI: 8.60–8.87) in males and from 10.06 (95 % CI: 9.96–10.15) to 12.00 (95 % CI: 11.85–12.16) in females (see Additional file 1).

## Discussion

The current study demonstrates that despite the availability of HZ vaccine, incidence of herpes zoster among an insured population has not decreased. A total of 113,871 immunocompetent patients with HZ were analyzed in this retrospective study. The overall annual HZ incidence rate for the year 2011 was 4.47 per 1,000 person-years in our dataset and 4.63 per 1,000 person-years when standardized for age and sex using 2010 US Census data.

Understanding the age-specific incidence rate of HZ is important as they represent the target population for zoster vaccine. Consistent with other studies, the risk of HZ increased with advancing age. The age-specific incidence ranged from 0.86 per 1000 person-years in individuals <19 years of age to 12.78 per 1000 person-years among those 80 years of age and older, and increased rapidly after 50 years of age. The age-related increase in the incidence may be closely linked to an age-dependent decline in VZV-specific cell-mediated immunity. However, the U.S. Food and Drug Administration approval of the zoster vaccine for use in adults over 50 years of age contrasts recommendations from the ACIP which recommends vaccination of adults 60 years and older [11]. A substantial public health burden of HZ also occurs in adults 50 to 59 years of age, most of whom are working adults. In our study, over 25 % of HZ occurred in adults 50 to 59 years of age and another 34 % occurred in adults >60 years of age in the US.

Our age- and sex-standardized estimated rate of 4.6 cases per 1,000 person-years among immunocompetent patients is comparable to recent studies using administrative data in the US. Previously, reported rates were 4.8 per 1,000 person-years from the general population that includes immunocompromised individuals in 2005–09 [8] and 4.4 per 1,000 person-years in 2006 [9]. Similarly, Yawn and colleagues reported incidence rates of 3.6 per 1,000 person-years in a population-based study in Minnesota that incorporated medical record review from 1996 to 2001 [5]. Another study which utilized the MarketScan databases, reported an incidence rate of HZ of 3.2 per 1,000 person-years in years 2000–2001 [7]. Studies conducted more than 20 years ago in the US showed even lower rates: 2.2 in years 1990–1992 [18] and 1.3 per 1,000 person-years in years 1945–1959 [19]. This trend over time is consistent with most studies that examined temporal trends in the US and six other countries across the globe [2, 9, 20–23].

We observed a higher incidence rate of HZ in women than men (5.2 versus 3.7 per 1,000 person-years, respectively) across all age groups. Although some studies found no difference, the majority of prior studies from across countries also found that women have a greater risk of HZ than men [1, 5, 20–22, 24–27]. This difference may be partly due to differences in health seeking behavior; however, the difference may also be due to biological factors. In the Shingles Prevention Study clinical trial where participants were actively followed up and cases were laboratory-confirmed, women had higher incidence rates of HZ than men in the placebo group (11.79 versus 10.65 per 1,000 person-years, respectively) [6]. It is plausible that there may be immunological or hormonal differences between women and men in response to reactivation of latent virus infection.

As with all retrospective studies, the current investigation has potential limitations. First, the study was limited to only those individuals with commercial health coverage or private Medicare supplemental coverage in the US; therefore, results may not be generalizable to HZ patients with other insurance or without coverage or those living outside the US. Second, the potential for misclassification of HZ, covariates, or study outcomes was potentially present as patients were identified through administrative claims data which are subject to data coding limitations and data entry error as opposed to medical records. However, previous studies found that the positive predictive value of ICD-9-CM codes for HZ in administrative claims data is relatively high, ranging from 84 to 94 % [18, 28, 29]. Third, since the zoster vaccine was approved in 2008 and this study only looked back 90 days prior to January 1, 2011 for evidence of vaccination, it is possible that both vaccinated and unvaccinated patients are included in the calculation. Fourth, this study used only one year of data to calculate incidence and may not represent the actual incidence if by any chance there were more or less cases reported in 2011. This study offers advantages, including the most updated estimate based on a nationwide administrative database in the US In addition, this study is based on a substantial number of datasets evaluated from two large administrative databases which allowed us to estimate the age- and sex-specific incidence rates in the immunocompetent population.

## Conclusions

Despite the availability of a vaccine, herpes zoster remains common among immunocompetent adults with incidence rates of HZ observed to increase with age and be higher in women than men. A significant focus on how to reduce the risk of HZ, e.g. improving vaccine access, should be adopted by health care providers.

## Additional file

Additional file 1:
**Incidence of Herpes Zoster among Immunocompetent Adults by Age and Gender.** This table contains the full set of incidence rates by age and by gender with confidence intervals. Please view this file as landscape. (DOC 45 kb)
