# Supplementary material for: Annual incidence rates of herpes zoster among an immunocompetent population in the United States
Source: BMC Infect Dis. 2015 Nov 6;15:502. doi: 10.1186/s12879-015-1262-8 (PMC4636742; doi:10.1186/s12879-015-1262-8)
Supplement: Additional file 1: — Incidence of Herpes Zoster among Immunocompetent Adults by Age and Gender. This table contains the full set of incidence rates by age and by gender with confidence intervals. Please view this file as landscape. (DOC 45 kb) [file 12879_2015_1262_MOESM1_ESM.doc]

Additional file 1: Incidence of Herpes Zoster among Immunocompetent Adults by Age and Gender

|  | Total population | | | | Men | | | | | Women | | | |
| --- | --- | --- | --- | --- | --- | --- | --- | --- | --- | --- | --- | --- | --- |
|  | Herpes zoster cases | Person-years | IR per 1000 person-years | (95% CI) | | Herpes zoster cases | Person-years | IR per 1000 person-years | (95% CI) | Herpes zoster cases | Person-years | IR per 1000 person-years | (95% CI) |
| Total | 113,861 | 25,466,740 | 4.47 | (4.45-4.50) | | 45,457 | 12,434,407 | 3.66 | (3.62-3.69) | 68,404 | 13,032,333 | 5.25 | (5.21-5.29) |
| Age Group | | | | | | | | | | | | | |
| <19 | 5,858 | 6,813,283 | 0.86 | (0.84-0.88) | | 2,881 | 3,476,311 | 0.83 | (0.80-0.86) | 2,977 | 3,336,972 | 0.89 | (0.86-0.92) |
| 20-29 | 7,727 | 2,820,528 | 2.74 | (2.68-2.80) | | 3,303 | 1,366,455 | 2.42 | (2.33-2.50) | 4,424 | 1,454,073 | 3.04 | (2.95-3.13) |
| 30-39 | 12,822 | 3,518,446 | 3.64 | (3.58-3.71) | | 5,383 | 1,683,271 | 3.20 | (3.11-3.28) | 7,439 | 1,835,175 | 4.05 | (3.96-4.15) |
| 40-49 | 19,181 | 4,242,104 | 4.52 | (4.46-4.59) | | 8,007 | 2,049,921 | 3.91 | (3.82-3.99) | 11,174 | 2,192,183 | 5.10 | (5.00-5.19) |
| 50-59 | 29,266 | 4,342,112 | 6.74 | (6.66-6.82) | | 10,507 | 2,096,846 | 5.01 | (4.92-5.11) | 18,759 | 2,245,266 | 8.35 | (8.24-8.47) |
| 60-69 | 21,595 | 2,317,241 | 9.32 | (9.20-9.44) | | 8,615 | 1,141,582 | 7.55 | (7.39-7.71) | 12,980 | 1,175,659 | 11.04 | (10.85-11.23) |
| 70-79 | 10,231 | 851,088 | 12.02 | (11.79-12.25) | | 4,223 | 400,085 | 10.56 | (10.24-10.87) | 6,008 | 451,003 | 13.32 | (12.99-13.66) |
| 80+ | 7,181 | 561,937 | 12.78 | (12.49-13.07) | | 2,538 | 219,936 | 11.54 | (11.09-11.99) | 4,643 | 342,001 | 13.58 | (13.19-13.96) |
| Combined age groups | | | | | | | | | | | | | |
| Age 50+ | 68,273 | 8,072,379 | 8.46 | (8.39-8.52) | | 25,883 | 3,858,450 | 6.71 | (6.63-6.79) | 42,390 | 4,213,930 | 10.06 | (9.96-10.15) |
| Age 60+ | 39,007 | 3,730,267 | 10.46 | (10.35-10.56) | | 15,376 | 1,761,604 | 8.73 | (8.59-8.87) | 23,631 | 1,968,664 | 12.00 | (11.85-12.16) |

CI, Confidence Interval; IR, Incidence Rate
